# Memeing scientific racism: the digital reframing of racialist ideologies

**DOI:** 10.1017/ehs.2026.10059

**Published:** 2026-06-17

**Authors:** Sarah Rodriguez-Louette

**Affiliations:** CREW, Universite Sorbonne Nouvelle, Paris, France

**Keywords:** racialism, scientific racism, memes, social media, strategies

## Abstract

This article examines how racialised memes circulating on X revive scientific racism, demonstrating that hybrid formats reconfigure biological essentialisation within contemporary digital culture. Using a corpus of 68 viral items and 4 case studies, our analysis applies Shifman’s content–form–stance model, informed by cognitive psychology. We show that these memes adopt common scientific conventions while relying on visual polarisation, whereby rapid perceptual contrasts stand in for arguments. Familiar templates, reinforced by selective algorithmic amplification, create a sense of perceptual legitimacy through fast categorisation and pre-attentive processing. We also trace how these cognitive shortcuts provide the basis for a regime of racialisation tailored to digitally immersed male audiences, producing gendered behavioural stereotypes and disappearance anxieties. A further pattern is the recoding of older racial hierarchies into neutral-sounding language of competence, allowing these ideas to travel across racialist, masculinist, techno-elitist, and anti-DEI (Diversity, Equity, Inclusion) communities. In this environment, memes work as micro-infrastructures of classification that align claims to objectivity with affect and platform incentives, making inequality appear natural and necessary. We conclude that effective critique must meet these images on their own terrain, with a pedagogy of the gaze that teaches publics how to recognise and challenge persuasion in meme-driven spaces.

## Graphical abstract

**Figure:**
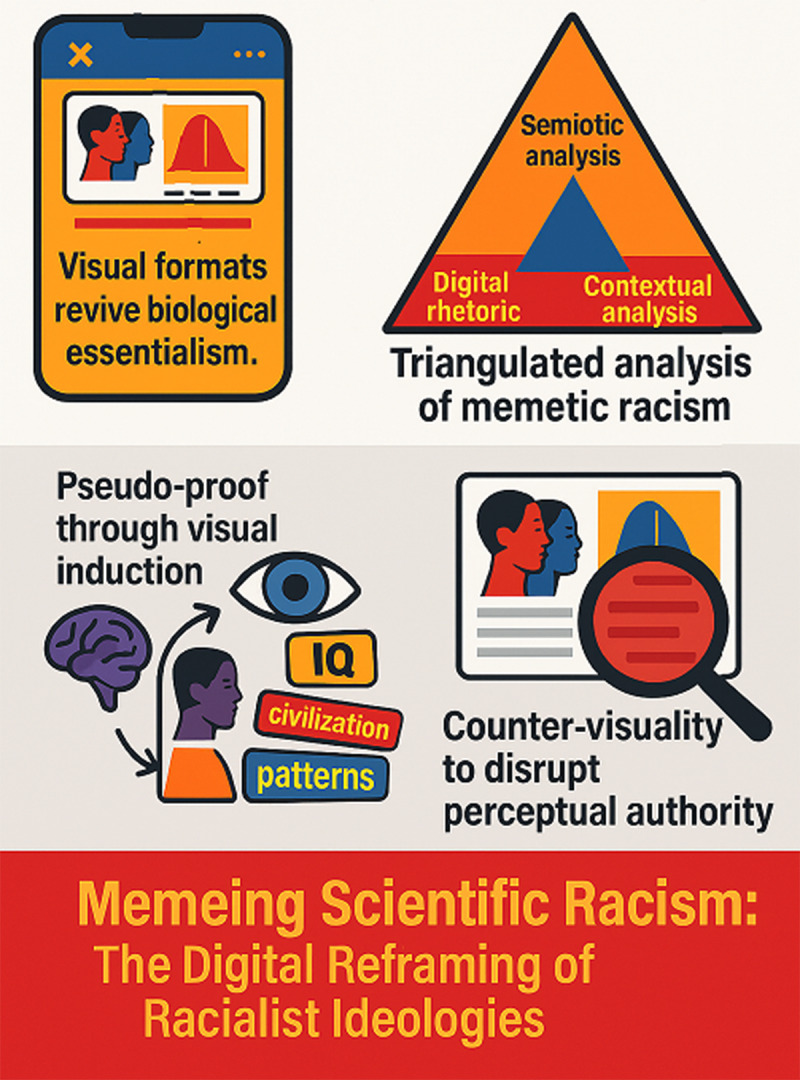

## Social media summary

Memes revive scientific racism by blending scientific aesthetics, primal-threat emotion, and transferable visuals to mask ideology.

## 1. Introduction

The slogan ‘One race, the human race’, circulated widely on social media, appears to affirm a universalist vision of humanity grounded in unity within diversity. Yet in digital settings, this ostensibly inclusive formula is often reworked through memes that mobilise caricature, schematic infographics, and decontextualised statistics to reintroduce logics of racial differentiation. Their seemingly playful format condenses hierarchical representations with a hybrid language that combines the aesthetic codes of scientific rationality with the emotional immediacy of internet humour.

Memes, defined as short visual–textual units circulated, imitated, and transformed by users (Shifman, 2013), derive their persuasive force from affective and perceptual modes that present themselves as self-evident, as opposed to relying on explicit argumentation. Their appeal lies in the capacity of iconic communication to condense complex meanings into readily apprehensible signs. By combining image, text, and familiar graphic conventions, memes mobilise well-documented cognitive shortcuts, including heuristic reasoning, processing fluency, and the dominance of intuitive appraisal over more deliberative evaluation (Reber et al., 2004; Tversky & Kahneman, 1974). In doing so, they exploit automatic visual processing in ways that can deflect critical scrutiny and adapt especially well to the attention-driven environment of contemporary platforms. When applied to racialised memes, these features work together to make hierarchical difference appear at once emotionally compelling and empirically grounded. Through repetition, salience, and rapid appraisal, racialised distinctions can come to seem familiar and commonsensical before they are subjected to more careful examination (Lee et al., 2024; Schwarz et al., 2021).

This phenomenon invites a broader historical perspective. The strategies deployed in racialised memes reactivate a long genealogy of scientific racism that, from the late eighteenth century onwards, sought to naturalise hierarchical difference through classificatory schemas, measurement of the body, and biological determinism (Painter, 2010). Early naturalist taxonomies such as those of Carl Linnaeus contributed to the fabrication of human ‘races’ as fixed categories, while nineteenth-century anthropometry, notably Samuel Morton’s polygenist craniometry, reinforced the belief that intelligence, morality, or civilisation could be inferred from anatomical indices. Although modern genetics has demonstrated that race lacks discrete biological boundaries, and social science has shown the structural rather than inherent nature of inequality (Smedley & Smedley, 2005), the epistemic structure of scientific racism persists. In contemporary self-described ‘race realist’ discourse, associated with figures such as Jared Taylor, social disparities are reframed as biological inevitabilities through selective invocations of IQ (Intelligence Quotient) statistics, simplified evolutionary narratives, and essentialised accounts of behaviour (Swain & Nieli, 2003).

A distinction must therefore be drawn between *social racism*, grounded in implicit stereotypes and everyday stigma (Lipsitz, 2018), and *scientific racism*, which legitimises hierarchical difference through ostensibly empirical evidence. As Gould (2008) argues, scientific racism operates through a regime of proof, mobilising measurements, typologies, and data visualisation to confer objectivity on value-laden judgements. In online environments, this logic leaves the pseudo-scholarly circles in which it once circulated and reappears in formats that blend emotional appeal with the visual authority of scientific representation. Memes compress this apparatus into compact images that recast classificatory hierarchies as everyday information within contemporary digital vernaculars.

Initially examined in subcultural ecosystems such as 4chan, Reddit, and Telegram (Nagle, 2017; Zannettou et al., 2018), racialised memes have often been read through the alt-right’s ironic style, in which détournement and transgression masked extremist commitments. Yet a focus on fringe spaces alone underestimates the extent to which these forms have migrated onto high-visibility platforms. Around the 2016 U.S. presidential election, particularly during the far right’s self-described ‘Great Meme War’, meme culture demonstrated its capacity to normalise extremist positions through participation and repetition (Bogerts & Fielitz, 2021).

Since Elon Musk’s acquisition of Twitter in 2022, and its subsequent rebranding as X, the platform has increasingly positioned itself as a ‘free speech’ environment, making racialised content more visible within mainstream digital culture (Rodríguez-Peral et al., 2025). Such memes now appear alongside posts by influencers, media outlets, and entertainment accounts, folded into a broader pop-cultural stream that softens their ideological charge even as it enhances their persuasive force. Presented as humour, evolutionary speculation, or ‘just data’, racial hierarchies can thus come to appear legitimate and ultimately be taken for granted.

It is against this backdrop, where long-standing epistemic structures intersect with platform-mediated visibility, that the present study interrogates the renewal of scientific racism in contemporary visual culture. Our aim is to examine how memes circulating on X reformulate the core tenets of scientific racism through the aesthetic, affective, and discursive conventions of digital media. More specifically, we address three questions:

1. How do racialised memes draw on anthropological and biological visual conventions to construct and stabilise racial categories under a veneer of scientific objectivity?
2. How do these memes employ affective and narrative cues that frame racialised out-groups as threatening, thereby facilitating rapid, intuitive responses?
3. How do they adapt references to concepts such as IQ, progress, or ‘civilisation’ to reproduce hierarchical interpretations of human variation within contemporary digital cultures?

To examine these dynamics, the article analyses a corpus of racialised memes collected from X through the analytical lens developed by Shifman, which focuses on the interplay between the content, form, and stance of memetic expressions (2013). This integrated approach makes it possible to trace how iconographic motifs, affective cues, and platform-specific modes of engagement converge to translate the epistemic logic of scientific racism into the cognitive and aesthetic environment of contemporary social media.

## 2. Methodology

### 2.1. Corpus and case selection

The corpus comprises 68 memes collected from X (formerly Twitter) between January and March 2025. Sampling followed a maximum-variation logic aimed at capturing the widest possible range of racialised meme formats, understood as recurrent combinations of visual structure and discursive framing. The objective was not to map the quantitative distribution of racialised content on the platform, but to identify the principal visual-discursive configurations through which scientific racism was being reframed during this period.

Sampling began with a high-visibility far-right account (over 400,000 followers at the time of collection), identified in prior research as a key aggregator of racialised and pseudo-scientific meme content within Anglophone nationalist online ecosystems (Rodriguez-Louette, 2024). From this entry point, a network of 30 interconnected accounts was delineated by tracing reciprocal interactions, supplemented by public profile information and observable following/follower patterns accessed through standard platform search and browsing features. This procedure mapped a broader far-right meme network while remaining fully compliant with X’s terms of service. These accounts exhibited diverse thematic foci, including religious polemics, antisemitic tropes, and anti-LGBTQ discourse, while only a core subset regularly posted racialised memes invoking biological or hierarchical claims. Notably, variations and exceptions emerged, making clear how these themes could be intermittently appropriated across adjacent ideological spaces.

Memes were selected according to two criteria. Firstly, high social visibility served as a proxy for circulation beyond fringe subcultures: only items that had received more than 5,000 likes at the time of collection were retained. To ensure accurate assessment of this threshold, the platform’s API (Application Programming Interface) was used to retrieve dates and engagement metrics for candidate posts from the selected accounts. Secondly, thematic relevance was evaluated through an examination of content, requiring either explicit references to biological difference and hierarchical claims, or implicit allusions such as templates, caricatures, or narrative scripts that evoked racial essentialism without naming it directly. Because X does not provide a dedicated image search function, the final corpus was assembled through targeted manual collection and verification. This hybrid approach, combining API-derived metrics for objectivity with manual curation for contextual depth and ethical handling, enabled precise identification of the most salient and representative racialised memes circulating online.

Each meme was documented with key metadata, including anonymised author, date, and number of likes, and coded inductively with attention to visual composition, textual content, and the emergent subject matter, which was then grouped into broader themes (Papapicco & Mininni, 2020). Recurring patterns aligned clearly with the study’s research questions, allowing straightforward assignment to three thematic categories: Essentialist Imagery (RQ1), Threat Imagery (RQ2), and Elitist Imagery (RQ3). Table 1 summarises the thematic distribution of the 68 memes, together with average engagement metrics and representative configurations.

**Table 1.** Thematic distribution of the corpus (*n* = 68) and average engagement metrics across themes. Average likes and views are calculated across all memes assigned to each thematic category 1 long description.

Theme	Frequency (n = 68)	Avg. likes	Avg. views	Configurations
*Essentialist Imagery*	31 (45.5%)	21K	1,389K	Facial juxtapositions
*Threat Imagery*	18 (26.5%)	33K	925K	Crime and demographic data
*Elitist Imagery*	19 (28%)	38K	1,181K	Civilisation timelines

From this corpus, four case studies were selected for their clarity in exemplifying distinct discursive configurations: (1) biological essentialism grounded in morphological contrast; (2) emotional threat narratives relying on behavioural stereotypes; (3) emotional threat narratives relying on demographic projections; and (4) civilisational decline framed through progress timelines. Although not exhaustive, these cases enable close examination of the multimodal strategies through which scientific-racist ideas are reformulated for digital audiences.

### 2.2. Ethical considerations

Given that the corpus includes harmful and demeaning imagery, ethical protocols emphasised harm minimisation and the careful handling of extremist content (Conway, 2021). Original memes are not reproduced in the article. Instead, the four case-study examples are presented exclusively through schematic reconstructions created using standard image-editing tools, including limited AI-assisted transformations. These reconstructions retain only general compositional features, such as the overall layout and the relative positioning of elements, while all offensive text, sensitive imagery, and any potentially identifying material have been removed. This approach allows the analytical structure of the meme to be conveyed without recirculating recognisable or politically charged imagery.

The corpus was analysed using a structured coding scheme. Memes from the wider corpus that inform the analysis but are not presented as case studies are referenced only through textual description, accompanied by an internal reference ID (M1–M68). All associated account data, including usernames, profile pictures, handles, and timestamps, were fully anonymised. The resulting dataset and its accompanying codebook are stored on a secure, access-restricted institutional server, where the original files are also preserved solely for verification purposes, in accordance with established guidelines for digital ethnography and visual research (Mertens & Ginsberg, 2009).

### 2.3. Analytical framework

The analysis combines a cultural framework grounded in Shifman’s work on internet memes with insights from evolutionary and cognitive psychology. Following Shifman (2013), memes are examined along three interconnected dimensions: content, form and stance. Content refers to the ideological and narrative themes conveyed by the meme, including essentialist claims about intelligence, civilisation or biological difference, as well as the causal stories and evaluative scripts through which these claims are organised. Form encompasses the iconographic and stylistic resources employed, such as infographics, comparative charts, evolutionary progressions, or physiognomic juxtapositions, together with recurring layout conventions, typographic choices, and genre templates that echo earlier pseudo-scientific traditions. Stance concerns the communicative orientation of the meme, describing how it positions the implied speaker and audience while situating the expression within a broader community or worldview.

Each meme in the corpus was examined through this integrative lens, with systematic notes taken on narrative patterns, compositional structure, and circulation patterns. Particular attention was paid to recurring motifs (for example, morphological contrasts, IQ maps, or progress timelines), to text–image relations (such as pseudo-statistical labels anchoring interpretations), and to the affective cues mobilised (fear, disgust, pride, or derision). This enabled the identification of patterns through which memes appropriate and update the visual repertoire of scientific racism.

In parallel, the analysis draws on work in evolutionary and cognitive psychology to clarify how these visual strategies interact with perceptual and affective biases. Research on in-group/out-group categorisation (Cosmides & Tooby, 1992), on threat sensitivity and error management heuristics (Haselton et al., 2015), and on intuitive, correlation-based reasoning (Kahneman, 2011) suggests that even minimalist formats can trigger essentialist interpretations of human variation. This is consistent with Brewer’s (1993) demonstration that minimal cues to social identity foster perceptions of distinctiveness and in-group homogeneity, reinforcing the tendency to essentialise group differences. The framework thus links cultural semiotics and cognitive predispositions, proposing that the persuasive force of racialised memes arises from their capacity to align inherited tropes of scientific racism with evolved tendencies in human information processing.

Drawing on the content–form–stance model, complemented by cognitive perspectives, this analytical approach addresses the study’s central question of how racialised memes circulating on X rearticulate key tenets of scientific racism within contemporary visual culture. It provides a consistent basis for examining the three dynamics developed in the results section: (1) the formal construction of pseudo-scientific authority; (2) the content-level shift from scientific to affective registers; and (3) the stance-related patterns that enable cognitive hierarchies to move across meme genres and reach wider digital publics.

## 3. Results

### 3.1. The image as a tool of racialisation: contrast and the illusion of science

#### The visual regime of racialist memes

In the contemporary digital environment, images function as a privileged vector of meaning: they circulate widely and condense ideological cues into perceptually fluent signals. On platforms such as X, this visual primacy is intensified by an attention-driven infrastructure that rewards contrast, salience, and emotional charge (Frau-Meigs, 2019). Memes epitomise this logic. Their compressed visual–textual format produces an immediate sense of legibility, generating an affective ‘click’ that feels like understanding while circumventing deliberation (Wiggins, 2019).

Across the corpus (*n* = 68), the most successful racialised memes rely on a simple but highly effective formula organised around the frontal juxtaposition of Black and White female faces in stark chromatic opposition. This pattern appears in 10 memes (15%), underlining its prominence. Posts structured in this way average around 23,000 likes, with one example (M40) exceeding 100,000, far above the few hundred likes typically garnered by non-visual racialist posts. These symmetrical pairings, generally set against neutral backdrops, isolate pigmentation as the dominant perceptual cue, making binary difference more immediately apprehensible than individuality, context, or contingency. Colour thus becomes a defining marker, converting a surface distinction into an essentialised social category. The repeated circulation of near-identical configurations further stabilises racial difference and renders it seemingly self-evident and readily recognisable.

This disproportionate prevalence cannot be explained by algorithmic dynamics alone. In visually saturated environments, sharply polarised compositions facilitate rapid categorisation, minimise interpretive complexity, and create the impression that meaning is available all at once. Their effectiveness relies on perceptual mechanisms that privilege categorical sorting through contrast and comparison. High-contrast arrangements are especially conducive to rapid uptake because visual perception is highly sensitive to marked differences in brightness, contour, and spatial separation: what stands out is registered first, often before deliberate interpretation begins (Haber & Hershenson, 1973).

Once faces are placed in frontal opposition and stripped of contextual detail, however, this perceptual economy is immediately socialised. Traits are apprehended through structured opposition and comparison, making difference appear intrinsic to the bodies displayed rather than contingent or contextual. Research in evolutionary psychology suggests that human cognition is especially responsive to distinctions legible in terms of affiliation and group boundary, including the contrast between in-group and out-group, between those perceived as belonging to ‘us’ and those marked as ‘them’ (Cosmides & Tooby, 1992). Interpretation therefore begins not from a neutral field of observation, but from a perceptual order already shaped by selection, simplification, and emphasis. By narrowing the field of attention to a small number of amplified signs, such memes allow racial meaning to take shape at the level of perceptual organisation before it is fully articulated discursively.

The specific use of the face as an object of social inference further intensifies this effect. Unlike statistical diagrams or DNA models, female faces trigger immediate social recognition and intuitive evaluation. Research on face perception shows that viewers rapidly infer traits such as trustworthiness, dominance, or warmth, while familiarity and attractiveness shape these impressions from the outset (Zebrowitz et al., 2007). The face thus functions less as a neutral representation than as a socially legible surface, one that invites assessment before reflection. Operating at the intersection of race and gendered visibility, these memes do not address a neutral observer so much as position the viewer to construct difference through an image already primed for social interpretation.

In this respect, racialist memes reactivate an older grammar of human classification. From François Bernier’s early groupings based on skin colour to anthropometric photography that presented pigmentation as a stable index of human type, chromatic differentiation long served to transform appearance into an apparently objective basis for categorisation (Sekula, 1986). This situated logic helps clarify why such memes constitute a first stratum of racialisation. As Omi and Winant (2015) argue, race is anchored not in biology itself but in a social regime of visibility in which bodily signs acquire meaning through cultural interpretation. Colourimetry, understood here as the strategic staging of chromatic opposition, functions within this regime as a representational device: through their formal simplicity, repeatability, and viral reach, such memes capture attention, polarise the visual field, naturalise distinction, and ultimately convert appearance into ideology.

#### Facial morphology and the reactivation of craniometric logics

**Figure 1.**
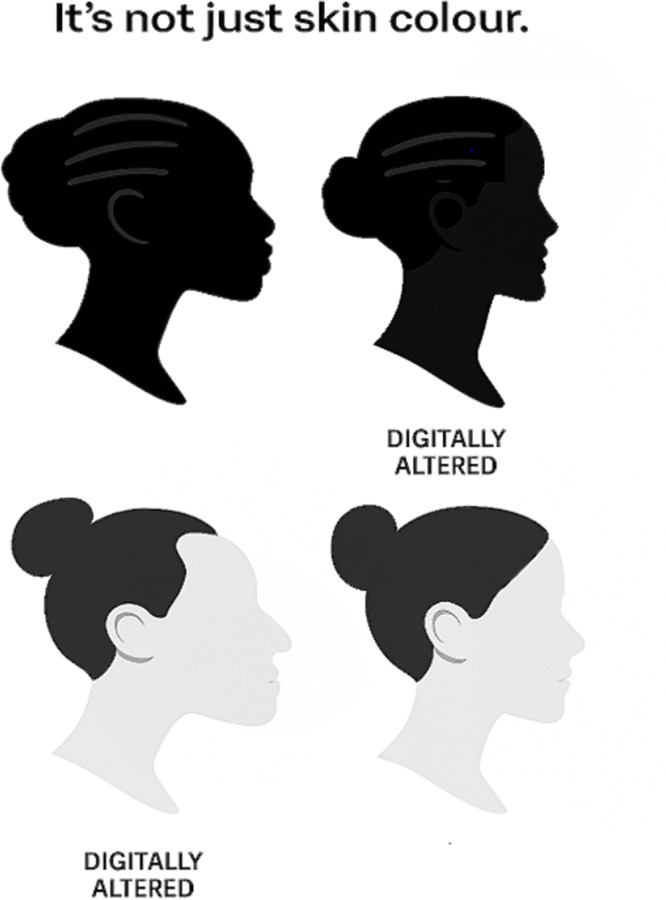
Schematic reconstruction of a meme juxtaposing Black and White female profiles. Digital alterations accentuate contrast while preserving only general compositional features.

A central meme in the corpus (M50, Figure 1) illustrates how digital culture reactivates older regimes of racial visualisation by shifting the focus from chromatic contrast to craniofacial structure. Organised in two rows and two columns, the image juxtaposes Black female faces (top) and White female faces (bottom), each shown in an original and a digitally altered version. The edits target features historically treated as morphologically diagnostic, especially forehead–jaw proportions, exaggerating or compressing cranial contours so that each face appears incongruous when reconfigured through the other’s features. The result is less an image of contrast than a graphic experiment in typological displacement.

The composition reproduces a familiar anthropometric aesthetic: neutral background, fixed profile angle, serial comparison, and symmetrical arrangement. Such conventions directly echo the grammar of racial science, from Blumenbach’s comparative heads to the standardised cranial photographs and anatomical plates used in nineteenth-century anthropology to render human difference measurable, comparable, and typological (Bhopal, 2007). Their function was never merely descriptive. These visual arrangements transformed bodily variation into classificatory evidence, presenting hierarchy as a matter of ordered appearance. The meme inherits this logic almost exactly: its formal neutrality creates the impression of detached observation while masking the ideological work of selection and exaggeration.

Seen in this light, the meme belongs to a longer epistemic history in which outward appearance was treated as the visible expression of inner human type. Early naturalist and anthropological taxonomies helped stabilise the idea that race could be read from bodily signs, especially cranial shape, facial angle, and other supposedly diagnostic markers (Painter, 2010). In such traditions, morphology did not simply describe the body; it *interpreted* it, making anatomy stand for intelligence, civilisation, temperament, and moral worth. What gave these images their authority was precisely their ability to collapse observation and explanation into a single act of looking. Contemporary memes abbreviate this legacy. They dispense with instruments, calipers, and measurement tables, yet preserve the core visual premise that group difference can be seen directly on the face and that comparison itself yields truth.

The caption ‘It’s not just skin colour’ functions as an anchorage (Barthes, 1977), steering the viewer towards the intended reading that racial difference lies in underlying morphological structure rather than superficial pigmentation. The montage’s persuasive force rests on a strategic asymmetry, as the Black face is subtly edited towards Eurocentric proportions, while the White face is altered towards features stereotypically coded as African. The effect is to produce an impression of structural irreducibility, encouraging the viewer to infer that each group possesses a distinct craniofacial type impermeable to overlap, variation, or historical contingency.

In doing so, the meme implicitly shifts the frame from socially constituted categories such as *Black* and *White* to the taxonomic imaginary of nineteenth-century racial typologies. Categories such as *Caucasoid* and *Negroid* were defined in naturalist classification systems as discrete, biologically bounded populations, each purportedly anchored in stable cranial and facial markers (Painter, 2010). Though scientifically obsolete, such terms persist because they promise to ground social difference in anatomy rather than in history or power. This is also what gives the meme a clear affinity with contemporary race-realist discourse, as circulated in public-facing form by Jared Taylor’s *American Renaissance*: visible morphology is treated not as one socially interpreted sign among others, but as evidence of enduring racial kinds. By foregrounding head shape as an index of group identity, the meme reintroduces morphology as a proxy for biological determinism.

Its ideological work therefore exceeds mere representation. Morphological contrast now performs the work once assigned to craniometric reasoning: individual variation is translated into categorical separation, and historically mediated distinctions are refigured as immutable biological boundaries. In this way, the meme updates the visual logic of scientific racism for a memetic environment, preserving the authority of comparative morphology while discarding the methodological procedures that once claimed to legitimate it.

#### Between scientific mimicry and platform validation

The ideological efficacy of racialised memes lies in their ability to reproduce the outward signs of scientific rationality while dispensing with its epistemic procedures. They do not test hypotheses, contextualise evidence, or submit claims to verification. Instead, they simulate objectivity by presenting ideological presuppositions as if they arose directly from neutral observation. This mechanism recalls what Gould (2008) identified in nineteenth-century scientific racism as a reversal of scientific reasoning: rather than deriving conclusions from empirical inquiry, classificatory materials were assembled so as to confirm pre-established hierarchies.

Contemporary memes inherit this structure. By selectively arranging visual cues, contrasts, and juxtapositions, they transform representation into apparent demonstration, making essentialised racial difference appear as something directly visible rather than interpretively constructed. This logic is especially visible in the circulation history of the photograph used in Figure 1 (M50). The image of the Black woman originally appeared in Afrocentric online contexts, where it was mobilised to suggest possible cultural and historical continuities between ancient Egypt and sub-Saharan Africa. In one such instance, it featured in a satirical composite captioned ‘How a Racist Meme Proved That Pharaohs Were Black’. The point was not to endorse the original racialist reading, but to invert it by exposing the instability of the visual claim. Yet a major racialist account in the dataset later reposted a screenshot of this composite without commentary (M31). Crucially, the repost neither rebutted the Afrocentric argument nor supplied counter-evidence. Its force lay precisely in silence. Detached from its original context, the image was made to function as an ironic cue, inviting viewers to treat the Afrocentric claim as self-refuting. What the image was taken to ‘prove’ therefore depended less on its content than on the interpretive dispositions of its audience.

This case shows that the persuasive force of such memes does not reside in visual form alone, but in the convergence of image, prior belief, and recognitional satisfaction. By compressing complex ideological cues into familiar and easily processed patterns, memes can generate a strong sense of fluency that feels epistemically satisfying. The act of ‘getting’ the reference or joke rewards recognition rather than scrutiny, reinforcing interpretations that already resonate with viewers’ expectations. Research on misinformation and persuasion suggests that material processed with ease is often experienced as more credible than analytically demanding claims, while repetition can turn familiarity into plausibility (Ecker, 2025; Wiggins, 2019). What appears persuasive, then, is less the strength of the argument than the feeling that the meme already makes sense.

The platform environment amplifies this process. Within an algorithmic economy of attention structured around salience, repetition, and engagement, images that polarise perception and confirm expectation are disproportionately rewarded (Gillespie, 2018). The reposted image (M31) accumulated more than 32,000 likes, more than three times the engagement of the original contrast-based meme (>9,000 likes). This disparity suggests that irony-driven circulation and decontextualised prompts can stabilise prior beliefs more efficiently than explicit argument. Likes and shares function here as social proof, generating a validation loop in which visibility stands in for correctness (Ecker, 2025). The meme’s claim is thus strengthened less by demonstration than by circulation itself: the more widely it is encountered, the more plausible it comes to seem.

Because these forms circulate within a mainstream digital arena rather than a confined extremist subculture, their effect exceeds the reinforcement of existing prejudice. They reactivate essentialist distinctions within a broader public sphere, embedding older racial taxonomies in ordinary habits of browsing, recognition, and sharing. Scientific racism is therefore not simply reproduced in memes; it is reconfigured within a perceptual-affective economy where cognitive ease, social validation, and algorithmic amplification converge to make ideological claims feel increasingly acceptable.

### 3.2. From science to emotion: behavioural stereotypes

#### Naturalising behavioural drives

Contemporary racialist memes no longer restrict themselves to essentialising morphology; they extend this essentialisation into the behavioural domain, presenting race as a psychological matrix. This shift is clearest in the recurring prompt to ‘notice patterns’, which functions less as a directive than as an interpretive cue. It signals that viewers should infer supposedly inherent dispositions (impulsivity, hypersexuality, violence, lack of empathy) attributed to non-white groups. These traits are rarely named. Instead, memes present mugshots, chaotic party scenes, or inflammatory tweets that encourage viewers to ‘connect the dots’ and treat behaviour as the visible trace of an internal racial nature.

A popular example encapsulates this logic (M43); a crow ‘recognising patterns’ is praised for intelligence, whereas a White male character ‘recognising patterns’ in a race-differentiated crime chart is condemned as racist. The humour rests on the insinuation that behavioural generalisations are empirical truths suppressed by social norms, recasting prejudice as observation and critique as censorship. What appears as a joke is in fact a thinly veiled argument that behavioural stereotypes are self-evident, requiring only the courage to acknowledge them. This reproduces, in digital mode, the core structure of contemporary race-realist theories. Psychologists such as Richard Lynn and J. Philippe Rushton argued that disparities in crime, income, or family structure reflected stable group differences in intelligence, impulse control, or empathy. Their framework replaced nineteenth-century craniometry with psychometric and behavioural indices while preserving the same causal chain, in which social inequality is attributed to inherited group dispositions rather than to structures of power. Racialist memes compress this logic into a portable vernacular form. Instead of psychological scales or regression tables, they present everyday vignettes that lead to the same conclusion: behaviour is treated as the expression of essence.

Numerical associations intensify the process of naturalisation. Rough graphs, simplified pie charts, or unreferenced histograms transform correlation into causation by foregrounding whichever representation maximises contrast. Even when absent, such formats continue to shape interpretation. Figures such as ‘13% of the population commits 50% of homicides’, drawn from the FBI’s Uniform Crime Report, circulate as mnemonic anchors (see M44). Their raw accuracy obscures how they collapse arrests, charges, and convictions and disregard well-documented biases in policing. Repeated without context, they generate informational saturation in which statistical association hardens into behavioural necessity. As Stuart Hall observed in *Policing the Crisis* (1978), statistics never function as neutral facts; they operate as narrative links. In racialist memes, they help weave a broader symbolic construction of behavioural danger. Numbers, visual cues, and pattern-recognition prompts converge to naturalise behaviour itself. The contingent becomes necessary, the historical becomes biological, and the social becomes psychological. Behavioural difference is staged as a prerequisite rather than a conclusion.

#### From data to alarm: embodying fear

If the data aesthetic lends an appearance of behavioural objectivity, a second stratum operates at a more immediate register, centred on the embodiment of threat. Here, danger is no longer inferred from numbers but materialised through screenshots, personal profiles and pseudo-documentary fragments that create an omnipresent interpretive backdrop. During corpus selection, many of the far-right accounts observed relied heavily on such materials (crime headlines, inflammatory comments, Tinder bios), which functioned as regular rhetorical devices rather than memes in a strict sense. Only one example (M41) had been sufficiently appropriated and recirculated to acquire meme status: an image of a White single mother with her mixed-race child, captured in an awkward moment and reused derisively across the network. These low-tech visuals draw on what Gillespie (2018) describes as the evidentiary authority of platform-native formats, in which the screenshot functions as a claim to authenticity irrespective of representativity or manipulation.

This surface realism masks a highly selective operation. The corpus relies on a constant accumulation of fragments, assembled less to document reality than to produce a climate of ambient threat. Each element is trivial in isolation; together, they create an intertext in which danger appears pervasive and embodied. What statistical memes insinuate, these snippets render visceral, mobilising both perceived realistic threats to safety and order and symbolic threats to racial and moral boundaries (Stephan & Stephan, 2000). Within this universe, the female form emerges as a central affective device, positioned at the crossing point of fear, sexuality, and moral judgement. Sometimes she appears as a victim – assaulted, murdered, ‘defiled’. Sometimes she is depicted as an accomplice – naïve, irresponsible, a propagator of dilution through miscegenation. The derogatory term *mudshark*, which circulates widely in these circles (Brindle, 2016), crystallises this double bind: the woman is simultaneously endangered and endangering. Her body becomes a symbolic hinge through which racial purity, social order, and sexual governance are negotiated.

**Figure 2.**
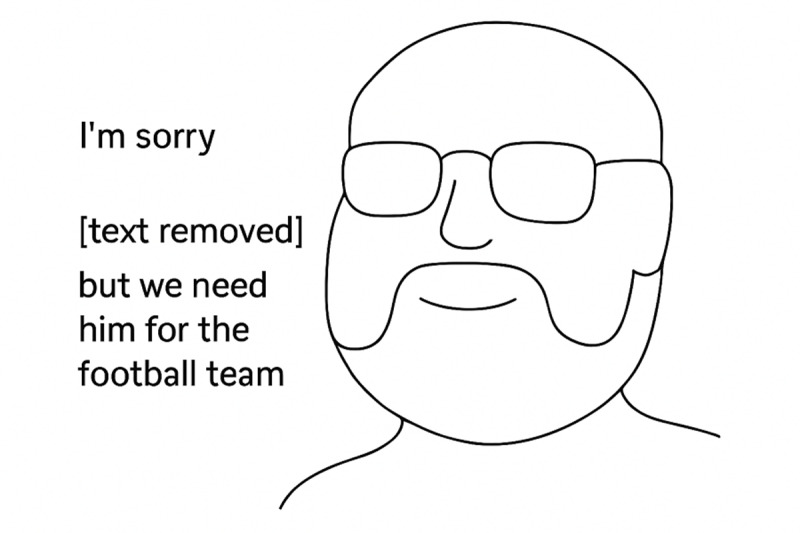
Schematic reconstruction of a meme using the ‘smuggie’ character to ridicule progressive positions.

Memes proper often emerge *after* this saturation of screenshots, functioning as distilled syntheses of the emotional narrative already established by dozens of decontextualised fragments. The soyjak example in Figure 2 (M60) exemplifies this dynamic. It addresses a father whose daughter has been ‘raped by a retarded cannibal’, yet insists that the perpetrator must be protected for the sake of a football team. The grotesque accumulation of violence, disability, and moral absurdity does not undermine the meme’s rhetorical force; it amplifies it, suggesting that tolerance is incompatible with group survival.

What interests the racialist meme is less plausibility than structure. The *soyjak* stands in for a caricatured progressive whose commitments conflict with self-preservation. Behind the absurd scenario sits the persistent racial typology of the hypersexual, predatory, uncontrollable Black male. This trope has deep historical roots, long consolidated across eugenic criminology, degeneration theory, and segregationist propaganda; it was systematised in the twentieth century by Richard Lynn and J. Philippe Rushton, who unified disparate stereotypes into a single pseudo-scientific persona of low impulse control, high aggression, and diminished empathy (Saini, 2019). Contemporary memes inherit and translate this persona into digital iconography.

Its resilience stems from the layering of registers. Crime, sexuality, pathology, and racial difference merge into a single cognitive gestalt that feels explanatory. This mechanism is akin to the automatic processes identified by Kahneman (2011), through which disparate cues are fused into a single, emotionally compelling interpretation. Once internalised, ironic exaggeration does not dilute the stereotype but instead facilitates its circulation. In this escalation from data anxiety to embodied alarm, racialised danger becomes both external (the threatening male body) and internal (the white woman whose choices imperil the group). Fear becomes the connective tissue that binds disparate visual elements into a coherent racial imaginary.

#### From group to species: disappearance and victimhood reversal

A third memetic register moves from behavioural essentialisation to demographic eschatology. Here, white populations are no longer presented as culturally dominant groups under pressure, but as biologically defined entities facing extinction. Memes crystallise this shift into compact formulae such as ‘ethnic/white genocide’ (M33, M46, M68) or ‘6%/white’ (M1, M67) that function as shorthand within a shared repertoire. These signals draw their force from an informational ecosystem where demographic facts, political grievances, and civilisational anxieties coalesce into a single narrative arc.

Central to this discourse is the recurrent reference to ‘6%’, derived from global projections suggesting that populations classified as White may constitute roughly 6–7% of the world population by the end of the twenty-first century (Chamie, 2024). Although rooted in United Nations datasets, these projections are decoupled from their determinants (fertility transitions, migration patterns, census reclassification) and become evidence of a biological contraction (Coleman, 2006). National projections are enlisted in the same way. The anticipated decline of the non-Hispanic White population in the United States to around 44% by 2060 (Vespa et al., 2020), or forecasts placing the White British population below 35% by 2100 (Goodwin, 2025), are mobilised as markers of an inexorable descent. Demography becomes teleology, and statistical change is read as an existential threat rather than sociological evolution.

**Figure 3.**
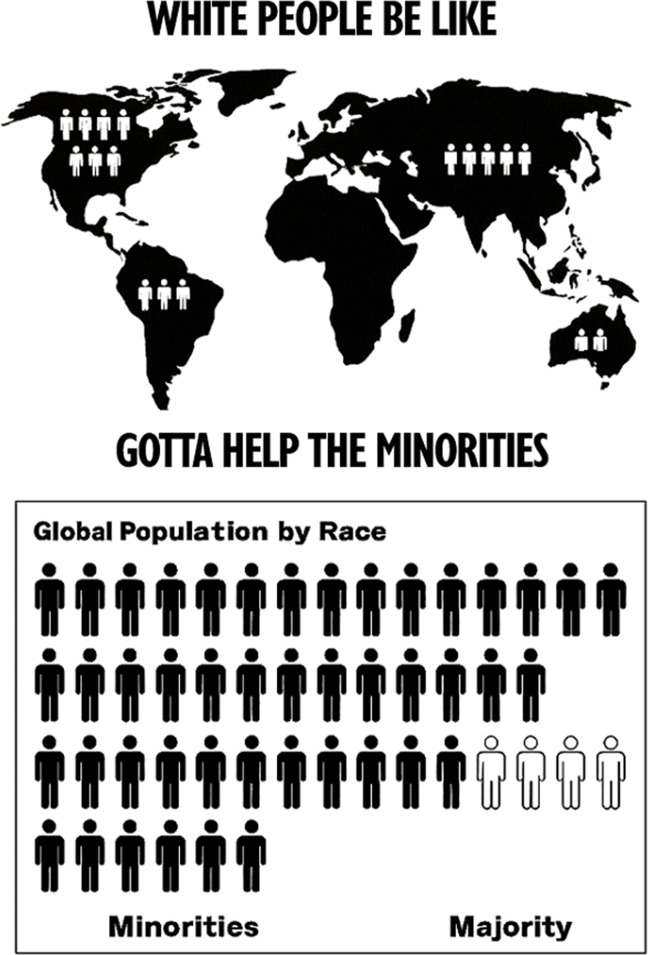
Schematic reconstruction of a meme combining a world map with stylised demographic icons.

Within this perspective, visual rhetoric plays a decisive role. A paradigmatic example is the meme captioned ‘Gotta help the minorities’, juxtaposing a colour-coded map of majority non-white regions with a crude global racial distribution diagram (M36, Figure 3). The composition is simple but rhetorically potent. By presenting Whiteness as a global minority, the meme performs a reversal of victimhood (Morris & Fredlund, 2025): minority-rights discourse is inverted so that the historically dominant group appears isolated, vulnerable, and morally entitled to protection. Whiteness is reframed not as a social position but as a demographically endangered species-like category.

This visual grammar operates through two complementary mechanisms. Firstly, it co-opts the codes of social justice graphics while inverting their ethical orientation. Secondly, it recasts diversity as an encroaching force. Spatial normality is displaced, and Whiteness is no longer imagined as the normative background of Western societies but as a shrinking enclave within an overwhelmingly hostile world. The shift from group to species collapses political identity into biological fate and transforms statistical trends into a race-against-time narrative in which disappearance appears inexorable. This eschatological orientation is reinforced by the layering of temporal registers. Dehistoricised nostalgia, present-day grievance, and projected extinction circulate concurrently within the same meme ecology. What begins as demographic change statistics culminates in an ontological narrative in which race itself becomes the primary unit of survival.

### 3.3. Cognitive hierarchy and memetic transversality: towards an anti-universalist coalition

#### Mainstreaming cognitive elitism

While psychometric charts still circulate in online racialist spaces, the corpus contains no explicit references to IQ. Instead, cognitive hierarchy is more often insinuated through euphemised, humorous formats. A meme featuring a relieved man and captioned ‘When Blacks start looting but you own a bookstore’ (M32) rests on the assumption that Black people are unlikely to read, establishing an implicit hierarchy of cognitive capacity without mentioning IQ at all. In this memetic economy, intelligence becomes a soft vector for racial differentiation. Instead of a measured variable, it is an ideological shorthand that positions some groups as cognitively deficient and others as inherently capable. This dynamic is striking given the scientific consensus. Research in cognitive psychology shows that IQ is not a fixed genetic trait but a plastic, environment-dependent construct shaped by socioeconomic conditions, schooling, nutrition, and discrimination (Nisbett et al., 2012). Yet memes circulate as if such consensus did not exist. Their persistence reflects ideological convenience rather than empirical coherence.

As with earlier racial typologies, the cognitive register belongs to a longer hereditarian lineage, extending from Francis Galton to Lewis Terman and Carl Brigham, ideologues who tied intellect to heredity and helped build the conceptual scaffolding later reactivated by late-twentieth-century racial hereditarianism. Richard Lynn and J. Philippe Rushton updated these frameworks by asserting stable, biologically rooted cognitive differences between human populations (Graves, 2002), though their influence remained largely confined to fringe milieus.

The decisive transformation came with Charles Murray. *The Bell Curve* (1994), co-authored with Richard Herrnstein, did not merely *revive* hereditarian arguments; it *mainstreamed* them. While the book has been extensively criticised for methodological flaws, for its genetic reductionism, and for construing social inequality as an inherited deficiency (Panofsky, 2014; Saini, 2019), it popularised the idea of cognitive stratification across racial, class, and gender groups. Crucially, Murray broadened the frame so that IQ functioned as both a racial signal and a generalised metric of cognitive worth, purportedly explaining economic and civilisational outcomes (Lemann, 1999). In memetic culture, this diffuse notion of IQ as naturalised worth circulates far more readily than any specific claim about genes or population differences. Two dynamics explain its renewed digital resonance.

Firstly, Murray’s expanded model maps onto contemporary anxieties about merit, competence, and institutional decline. Because it extends beyond race to class, gender, and ‘undeserving’ groups, it offers a general grammar of hierarchy well-suited to culture-war conflicts. Secondly, cognitive insinuation now folds into anti-DEI rhetoric. A meme captioned with the pun ‘Vote DEI, you die’ (M21) juxtaposes wildfire images with a White and a Black female official, insinuating that ‘diversity hires’ are incompetent. Posted by a major far-right influencer, not a racialist account, the meme illustrates how IQ-coded insinuations now circulate across mainstream right-wing publics. Engagement metrics confirm this broad circulation, with the meme garnering over 7 million views and 135,000 likes, far surpassing all other items in the corpus. As Daniels (2018) notes, this ludic ambiguity enables layered reception: literal for the initiated, ironic for sceptics of universalism, and shareable for mainstream anti-progressive audiences. The plasticity of uptake is precisely what allows cognitive hierarchy to persist as a meaningful structure while shedding the discursive markers of explicit racialism.

#### The meme of civilisational decline

At the margins of the memetic universe, references to cognitive hierarchy circulate as weak yet persistent signals. They surface in sarcastic remarks about competence so as to become a transversal operator linking otherwise distinct ideological spaces, enabling racialised meanings to circulate in ostensibly meritocratic terms.

**Figure 4.**
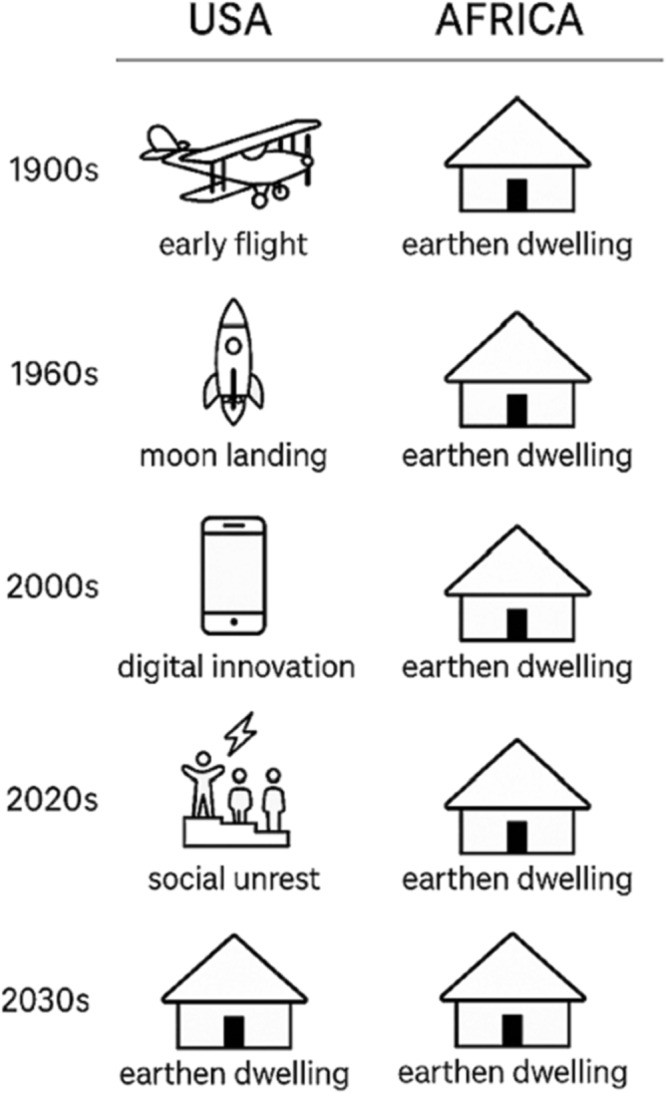
Schematic reconstruction of a meme contrasting the technological history of the United States with a stereotyped image of an earthen dwelling used to symbolise Africa.

Humour in this economy quickly shifts into a darker affective register aligned with black-pill fatalism. The objective shifts from laughter to alarm. A paradigmatic example sets the technological history of the United States (early aviation, the Moon landing, Silicon Valley) against a recurrent image of a dwelling presented as emblematic of Africa (M12, Figure 4). In the final panel, set in the 2030s, the dwelling appears on American soil, allegedly imported through immigration in the 2020s. The implied slogan, ‘Import the Third World, become the Third World’, recasts racial diversity as civilisational contamination.

The rhetorical mechanism hinges on graphic juxtaposition that invites causal inference. The African continent is depicted as the negation of progress, its proximity signalling the onset of modernity’s collapse. Cognitive biases, such as the correlation illusion, encourage viewers to read co-occurrence as causality (Haselton et al., 2015). In this montage, ‘Africa’ functions as a symbolic placeholder for stagnation, crystallising a tripartite ideological structure: a dichotomy between innovation and regression; an attribution of inventive capacity to ‘Whiteness’ via icons such as the Wright brothers, Armstrong, or Jobs; and a reversal in which the continent, once externalised as the antithesis of progress, returns as an invasive force jeopardising the West. This visual grammar revives long-standing colonial stereotypes portraying Africa as ahistorical and incapable of development. It draws directly on the ideological repertoire of early twentieth-century scientific racialism, notably the work of Madison Grant and Lothrop Stoddard, for whom race was taken to determine a civilisation’s capacity for innovation and diversity to signal decline (Saini, 2019). Contemporary far-right cultures, including post-alt-right iterations, have revived Grant and Stoddard’s narratives as explanatory models for present-day demographic change (Berbrier, 2018; Hawley, 2017). The meme distils this worldview into a minimalist, iterative montage, replacing argument with inductive suggestion tailored to digital cognition.

Such a symbolic structure resonates with contemporary political debates on immigration and identity. By visualising demographic change as an existential threat to technological modernity, the meme organises political anxieties into a coherent narrative template. It circulates widely in communities that already define policy reform as defensive necessity, providing ideological scaffolding for calls to restrict immigration or to preserve a putatively endangered civilisational core. The iconographic choices are deliberately calibrated to reach far beyond explicitly racialist circles. Engineers, inventors, explorers, entrepreneurs, and figures venerated within innovation-centric cultures serve as positive metonyms of pioneers. This encodes racial hierarchy in a vocabulary attuned to audiences who interpret social issues through productivity, merit, and technological achievement. The meme thus finds natural resonance on the Musk-era X platform, where anti-DEI sentiment converges with a techno-optimist ethos that celebrates productive ‘builders’ and imagines engineering talent as better spent on grand civilisational projects such as space colonisation (Vinsel & Russell, 2020).

By staging civilisational change as a threat to innovation itself, the meme translates a racial hierarchy into a rhetoric of efficiency and cognitive output. Its quasi-documentary aesthetic masks the deeply ideological claim that inequality reflects inherent, immutable differences rather than historical or structural conditions. This euphemised register is further developed in online variants of Human Biodiversity associated with writers such as Steve Sailer, where terms like ‘population differences’ replace explicit talk of race while preserving the same naturalising logic (Panofsky et al., 2020). Such implicit naturalism is emblematic of Scientific Racism 2.0, in which classical racial hierarchies are recoded into ostensibly post-ideological, techno-scientific terms suited to the sensibilities of contemporary digital publics.

#### Towards an anti-universalist elitist coalition

The network of 30 accounts examined displays a stratified configuration consistent with previous research on online infrastructures of ideological propagation (Rodriguez-Louette, 2024). One highly active account operates as an ideological aggregator, centralising and recycling explicitly racialist material. A secondary relay initiates or amplifies these posts, maintaining discursive continuity. Three prominent far-right accounts linked to non-racialist profiles adopt a more opportunistic strategy, contributing six memes to the corpus and circulating cognitive or civilisational tropes.

Together, these memes delineate a shared ideological language within the contemporary X ecosystem, reshaped since 2022 by Elon Musk’s libertarian governance and the consolidation of a harder-edged digital right. Despite their surface heterogeneity, the accounts converge in rejecting universalism and elevating cognitive superiority as a political criterion. The Dark Enlightenment, whose foundations were laid by Curtis Yarvin in the late 2000s, provides a conceptual matrix for this neo-reactionary convergence. It rejects democracy and egalitarianism in favour of hierarchical authority, casting elites as the natural drivers of civilisational progress (Taşkale, 2025). Closely aligned with Silicon Valley’s individualism, this milieu presents egalitarian commitments as obstacles to innovation and portrays democracy as structurally inefficient.

In later appropriations of these ideas, IQ is deployed less as a psychometric tool than as a political marker, reinstating hierarchy under the ostensibly red-pilling language of statistical revelation (Aikin, 2019). Memes function here as vehicles of ideological cross-pollination: whether ridiculing looters’ supposed illiteracy or predicting decline through tropes of racialised underdevelopment, they activate the same cognitive frame, one in which social outcomes are explained by innate capacities. What once appeared as explicit racial hierarchy is rearticulated through the ostensibly neutral language of competence, productivity, and ‘cognitive capital’, enabling audiences as diverse as racialists, techno-elitists, anti-DEI activists, and masculinist communities to locate themselves within a shared evaluative logic.

In this way, memes circulate ideology across disparate publics by producing forms of compatibility and condensing hierarchical imaginaries into shared symbolic units. They rebrand racialised hierarchies as matters of optimisation or civilisational momentum and thereby provide the symbolic infrastructure for an emerging anti-universalist, elitist coalition organised around competition and the repudiation of egalitarian norms.

## 4. Discussion

### 4.1. The mimetic reactivation of scientific racism: perception as proof and prescription

The findings indicate a strong formal continuity between the visual devices of nineteenth-century scientific racism and the image-based racialisation strategies circulating on X. As in anthropometric plates, comparative typologies, or early craniometric photography, the memes analysed here employ standardised poses, frontal or profile symmetry, and the isolation of salient traits to create an impression of legibility. Yet, whereas historical racial science sought authority by combining measurement and visualisation (through indices, grids, or calibrated photographs), the contemporary meme relies exclusively on perceptual induction. It imitates the stylistic codes of scientific observation (neutral backgrounds, comparable angles, schematic contrast) while detaching them from empirical procedure. In this sense, the meme generates the perception of evidence, producing conviction through immediacy.

Existing scholarship on misinformation and persuasion (Ecker, 2025; Wiggins, 2019) has shown how perceptual fluency can be mistaken for evidential strength. The present analysis extends this insight by demonstrating that such fluency is achieved through the revival of scientific-racist conventions. What once required instruments, calibrations, or typological plates is now reproduced through compressed, standardised templates optimised for platform circulation. This mimicry produces what may be termed *perceptual legitimacy*. It complements, but also complicates, accounts of ‘data realism’ or ‘statistical persuasion’ in digital extremism (Tuters & de Zeeuw, 2020). Rather than relying on numbers or graphs, high-engagement memes mobilise perceptual cues that circumvent deliberation altogether. Scientific racism is therefore not simply reproduced; it is reactivated as a vernacular epistemology, one that deploys the authority of the visual to reassert essentialist distinctions within contemporary digital culture.

The epistemic effects of this visual regime extend directly into the political domain. When racial difference is rendered perceptually manifest, the boundary between demographic prognosis and political prescription narrows. As Swain (2004) has shown in relation to White nationalist discourses, once difference is posited as natural and immutable, separation can be framed as rational necessity rather than ideological extremity. The memes analysed here enact precisely this shift. By repeatedly staging White populations as demographically vulnerable or existentially threatened, they naturalise the idea that coexistence is untenable and that social and political boundaries must be redrawn accordingly (Rodriguez-Louette, 2025).

The novelty of the present analysis lies in demonstrating how this political orientation is produced compositionally. Dominant meme formats (two-panel contrasts, face-to-face juxtapositions, dichotomous progressions) encode opposition at the level of iconographic structure. These binary layouts restrict interpretation to mutually exclusive categories, erasing gradation or overlap and thereby creating a perceptual environment in which racial separation appears not only plausible but structurally inevitable. Where the literature on polarisation has emphasised affective reinforcement, algorithmic sorting, or ideological echo chambers (Bail, 2021), these findings highlight a complementary mechanism: *visual polarisation*, emerging less from discourse than from the perceptual constraints of the images themselves. The meme thus functions as a micro-infrastructure of classification, aligning epistemic claim (proof of difference) with political prescription (the ordering of populations through separation) within the same binary framework.

### 4.2. Algorithmic racialisation and the gendered construction of threat

The corpus demonstrates that racialised memes derive persuasive force from the interaction of cognitive heuristics, platform infrastructures, and gendered frames of perception. Whereas prior research has emphasised ideological content or affective antagonism (Daniels, 2018), the present analysis shows that their efficacy often precedes explicit belief formation. These memes work by making racialised interpretations feel socially legible and affectively coherent before they are fully articulated as doctrine.

At a first level, memes activate classical cognitive shortcuts such as rapid pattern recognition, correlation illusion, and base-rate neglect, predisposing viewers to infer patterned regularities even from incomplete or selectively curated data (Haselton et al., 2015; Kahneman, 2011). Screenshots, mugshots, and visual snippets acquire explanatory force because they fit intuitive causal templates grounded in salience and contrast, catalysing essentialist inference (Cosmides & Tooby, 1992). Yet heuristics alone cannot explain the speed with which such interpretations consolidate on X. Algorithmic infrastructures amplify contrast-rich and emotionally arousing content, creating a visibility economy in which repetition functions as an epistemic cue, engagement metrics as social proof (Ecker, 2025), and recurring formats as norm cues within networked publics. In this way, algorithmic curation contributes directly to the stabilisation of racialised associations, aligning with research on the role of digital platforms in shaping norms, threat perception, and political interpretation (Barberá, 2020).

This dynamic becomes especially visible where race, gender, and sexuality converge. The persuasive power of such memes rests on their production of self-evidence: unlike statistical diagrams or DNA models, faces and bodies trigger immediate social recognition and intuitive evaluation. Research on face perception shows that viewers rapidly infer traits such as trustworthiness, dominance, or warmth, while familiarity and attractiveness modulate these impressions (Zebrowitz et al., 2007). The body thus becomes a visual argument, a surface that appears legible before reflection. Memes presuppose a male spectator and mobilise recurring tropes of racialised threat, miscegenation anxiety, and the policing of White femininity. In this configuration, sexual valuation and racial categorisation become inseparable: White femininity is rendered desirable, vulnerable, and in need of protection, while Black bodies are coded through danger, contamination, or excess. Biological essentialism thus operates across both race and gender (hooks, 2015), making women central to a broader affective script in which sexuality, reproduction, purity, and decline are fused into a single narrative of threat.

Taken together, these mechanisms create a compound regime of racialisation tailored to digitally immersed male publics. The force of these memes lies both in ideological content and in their capacity to stack otherwise distinct anxieties (criminal, sexual, demographic, and civilisational) into a format that is cognitively fluent, emotionally resonant, and socially shareable. Algorithmic racialisation is therefore not merely the amplification of prejudice, but the construction of a receptive field in which racialised meanings appear intuitive, collectively legible, and visually anchored.

### 4.3. The meme as a transversal language of anti-universalism

A further implication of these findings is the emergence of a shared ideological grammar that cuts across otherwise heterogeneous right-wing digital publics. On X, under techno-libertarian governance, racialist memes appear alongside the discourses of techno-optimists, masculinist influencers, and anti-DEI commentators. These actors do not form a unified movement, but they nonetheless coalesce around a rejection of egalitarian universalism and a recurrent elevation of cognitive superiority as a political and moral criterion. The meme form plays a central role in this alignment, recoding hierarchical claims through ostensibly neutral categories such as merit, productivity, and optimisation, and enabling disparate groups to recognise their concerns within a shared evaluative framework. Such idioms circulate easily across broad reactionary communities because meritocratic narratives render them both intelligible and legitimate, ultimately presenting hierarchy as a naturalised order whose contestation appears socially corrosive.

Within this environment, the meme functions as a cultural interface, translating differentialist worldviews into a lightweight, humorous format that can be taken up across otherwise distinct publics. Through repetition and aesthetic simplification, it normalises hierarchical imaginaries within everyday digital culture, making them both intelligible and socially portable even in domains that do not explicitly identify as racialist.

However, this contemporary shift extends the logic of Francis Galton, who conceived intelligence as hereditary capital optimisable through selective intervention (Aubert-Marson, 2009), into a twenty-first-century imaginary. Echoes of this logic reappear in transhumanist visions, optimisation-oriented strands of Effective Altruism, and broader cultural valorisations of cognitive rationality as a measure of social worth. Crucially, the emphasis moves beyond racial segregation towards patterns of intra-racial eugenic stratification, in which those deemed weak or unproductive are marked as impediments to collective advancement. What emerges is a reconfigured hierarchy in which elitist differentiation exceeds explicit racialism and becomes embedded in broader techno-cultural projects of optimisation and selective enhancement.

### 4.4. Unpacking the politics of evidence: towards a situated visual critique

The memetic reworking of racial hierarchy described above poses a challenge for conventional critical strategies. Scientific rebuttal and moral denunciation have a limited effect on a discursive mode that presents itself less as a proposition to be evaluated than as a prompt appealing to intuition rather than deliberation. In this register, the meme’s authority is grounded in suggestion, repetition, and the tacit plausibility generated by platform infrastructures. What is encountered is not simply a thesis to refute, but a felt truth installed through perceptual fluency.

A critical response must therefore attend to the conditions of seeing that allow these representations to function as evidence. Three avenues appear especially necessary. Firstly, counter-visual strategies that disrupt the aura of neutrality through juxtaposition, contextualisation, or situated parody can expose the constructed nature of racialist cues. Secondly, systematic re-anchoring of data, images, and screenshots is needed to counteract the atemporality of memetic evidence. In practice, this means reconnecting decontextualised crime screenshots, demographic graphs, or facial comparisons to the institutional, temporal, and statistical conditions from which they have been detached. Thirdly, a pedagogy of the gaze, attentive to visual grammar, cognitive bias, and algorithmic amplification, offers a way to re-politicise perception itself. Such an approach has implications for media literacy, digital citizenship, and educational settings in which students increasingly encounter political claims through memes.

A platform-specific perspective helps clarify how these dynamics take shape. On X, the frictionless circulation of screenshots, metrics, commentary, and image macros collapses evidentiary, ironic, and insinuative registers into a single public stream, enabling racialised claims to shift rapidly between humour, proof, and political signalling. On more visually immersive platforms such as TikTok or Instagram, similar hierarchical imaginaries would manifest through aestheticised or audio-visual cues (short-form video, influencer performance, and ambient lifestyle signalling) rather than screenshot-based insinuation. Across these environments, the challenge is compounded by the fact that such claims are embedded in images, composites, and hybrid formats that remain far more difficult for automated moderation systems to detect and interpret than explicit textual statements.

More broadly, the meme has become a site of epistemic struggle, where questions of evidence, difference, and hierarchy are reworked through a tailored visual vocabulary. What once circulated as anthropometric ‘science’ now appears as light, shareable micro-content optimised for digital diffusion. Within this perceptual-affective economy, older racial taxonomies are reactivated less as formal doctrines than as viral intuitions embedded in everyday browsing, recognition, and sharing. By foregrounding the visual, affective, and infrastructural mechanisms through which inequality is naturalised, this study argues that critique must intervene on the same terrain (visual, memetic, algorithmic) where racialised claims acquire traction. What is required is a situated critical anthropology of digital imagery, attuned to modes of conviction that emerge not through argument but through the immediacy of the glance.

## Supporting information

10.1017/ehs.2026.10059.sm001 Rodriguez-Louette supplementary material Rodriguez-Louette supplementary material

## Supplementary material

The supplementary material for this article can be found at https://doi.org/10.1017/ehs.2026.10059.

## Research transparency and reproducibility interest

The data used in this study consist of 68 anonymised memes collected from social media platforms. In compliance with privacy concerns, any identifiers or traces of the accounts that shared the memes have been removed. These memes can be accessed for consultation via a dedicated server or, where appropriate, through platform links after publication. Access will be provided by the corresponding author in accordance with the journal’s data-sharing guidelines.

## Appendix: Key cognitive and perceptual terms in meme contexts

**Table:**

Term	Definition	Meme cues
*Base-rate neglect*	Ignoring general statistics in favour of vivid examples	Anecdotal memes presented as evidence
*Category salience*	Certain categories stand out more in perception	Labels, clustering, exaggerated group markers
*Confirmation bias*	Favouring information that fits prior beliefs	Cherry-picked screenshots, partisan compilations
*Emotional salience*	Strong emotional content grabs attention and feels more important	Crime overlays, outrage captions
*Familiarity effect*	Repetition makes information feel true	Templates, slogans, viral formats
*Induction*	Generalising from a few cases	‘One example proves the rule’ memes
*Illusory correlation*	Seeing links where none exist	Selective stats implying group traits
*Perceptual legitimacy*	Scientific-looking visuals feel credible	Charts, graphics, references
*Perceptual fluency*	Easy-to-process visuals seem more believable	Bold contrasts, simple layouts, clear fonts
*Pre-attentive processing*	Automatic perception of basic features before conscious thought	Colour coding, arrows, bold highlights
*Rapid visual categorisation*	Immediate sorting of images into social types	Side-by-side faces, contrasts, oppositions
*Social proof*	Popularity signals imply correctness	Likes, reposts
*Threat perception bias*	Overestimating danger from stereotyped groups	Dark filters, warning icons

## Table 1 Long description

The table summarizes three thematic categories in a 68-item corpus, reporting how often each theme appears, average likes, average views, and the typical content configuration used. Essentialist imagery is the most frequent theme with 31 items, followed by elitist imagery with 19 and threat imagery with 18. Average likes are highest for elitist imagery at 38 thousand, then threat imagery at 33 thousand, and lowest for essentialist imagery at 21 thousand. Average views are highest for essentialist imagery at 1,389 thousand, followed by elitist imagery at 1,181 thousand, with threat imagery lowest at 925 thousand. The configurations differ by theme: essentialist uses facial juxtapositions, threat uses crime and demographic data, and elitist uses civilisation timelines. These averages are calculated across memes assigned to each theme, so they describe typical engagement within categories rather than totals across the corpus.

Navigate back to Table 1.
