# Supplementary material for: Memeing scientific racism: the digital reframing of racialist ideologies
Source: Evol Hum Sci. 2026 Jun 17;8:e27. doi: 10.1017/ehs.2026.10059 (PMC13358990; doi:10.1017/ehs.2026.10059)
Supplement: Rodriguez-Louette supplementary material [file S2513843X26100590sup001.docx]

**Appendix: Key Cognitive and Perceptual Terms in Meme Contexts**

| **Term** | **Definition** | **Meme cues** |
| --- | --- | --- |
| **Base‑rate neglect** | Ignoring general statistics in favour of vivid examples | Anecdotal memes presented as evidence |
| **Category salience** | Certain categories stand out more in perception | Labels, clustering, exaggerated group markers |
| **Confirmation bias** | Favouring information that fits prior beliefs | Cherry‑picked screenshots, partisan compilations |
| **Emotional salience** | Strong emotional content grabs attention and feels more important | Crime overlays, outrage captions |
| **Familiarity effect** | Repetition makes information feel true | Templates, slogans, viral formats |
| **Induction** | Generalising from a few cases | “One example proves the rule” memes |
| **Illusory correlation** | Seeing links where none exist | Selective stats implying group traits |
| **Perceptual legitimacy** | Scientific‑looking visuals feel credible | Charts, graphics, references |
| **Perceptual fluency** | Easy‑to‑process visuals seem more believable | Bold contrasts, simple layouts, clear fonts |
| **Pre‑attentive processing** | Automatic perception of basic features before conscious thought | Colour coding, arrows, bold highlights |
| **Rapid visual categorisation** | Immediate sorting of images into social types | Side‑by‑side faces, contrasts, oppositions |
| **Social proof** | Popularity signals imply correctness | Likes, reposts |
| **Threat perception bias** | Overestimating danger from stereotyped groups | Dark filters, warning icons |
